# A polymorphism in the dysbindin gene (*DTNBP1*) associated with multiple psychiatric disorders including schizophrenia

**DOI:** 10.1186/1744-9081-6-41

**Published:** 2010-07-09

**Authors:** Joanne Voisey, Christopher D Swagell, Ian P Hughes, Jason P Connor, Bruce R Lawford, Ross M Young, C Phillip Morris

**Affiliations:** 1 Institute of Health and Biomedical Innovation, Queensland University of Technology, Brisbane, Queensland, Australia; 2 Discipline of Psychiatry, School of Medicine, The University of Queensland, Herston, Queensland, Australia; 3 Division of Mental Health, Royal Brisbane and Women's Hospital, Brisbane, Queensland, Australia

## Abstract

**Background:**

A number of studies have found associations between dysbindin (*DTNBP1*) polymorphisms and schizophrenia. Recently we identified a *DTNBP1 *SNP (rs9370822) that is strongly associated with schizophrenia. Individuals diagnosed with schizophrenia were nearly three times as likely to carry the CC genotype compared to the AA genotype.

**Methods:**

To investigate the importance of this SNP in the function of *DTNBP1*, a number of psychiatric conditions including addictive behaviours and anxiety disorders were analysed for association with rs9370822.

**Results:**

The *DTNBP1 *polymorphism was significantly associated with post-traumatic stress disorder (PTSD) as well as nicotine and opiate dependence but not alcohol dependence. Individuals suffering PTSD were more than three times as likely to carry the CC genotype compared to the AA genotype. Individuals with nicotine or opiate dependence were more than twice as likely to carry the CC genotype compared to the AA genotype.

**Conclusions:**

This study provides further support for the importance of *DTNBP1 *in psychiatric conditions and suggests that there is a common underlying molecular defect involving *DTNBP1 *that contributes to the development of several anxiety and addictive disorders that are generally recognised as separate clinical conditions. These disorders may actually be different expressions of a single metabolic pathway perturbation. As our participant numbers are limited our observations should be viewed with caution until they are independently replicated.

## Background

Dystrobrevin binding protein 1 (DTNBP1) is a neuronal protein that binds to alpha- and beta-dystrobrevin in muscle and brain [1]. Association and functional studies suggest a role for the *DTNBP1 *gene in schizophrenia. Linkage studies first identified *DTNBP1 *as a region of high schizophrenia susceptibility [2-4] and polymorphisms in *DTNBP1 *have since been identified to be associated with schizophrenia [3,5,6]. Biologically, *DTNBP1 *is a strong candidate for schizophrenia pathogenesis as it is thought to play a pivotal role in regulating the glutamatergic system [7,8]. Glutamate hypofunction leads to increased sensory flooding and changes in dopamine concentration. Hence, glutamate receptor agonist drugs are now used in the treatment of schizophrenia [9]. Functional studies of *DTNBP1 *provide further evidence for its role in schizophrenia aetiology. Reduced *DTNBP1 *mRNA expression in cerebral cortex has been associated with risk haplotypes for schizophrenia [10]. Reduced *DTNBP1 *mRNA and protein expression has been found in the hippocampal formation and dorsolateral prefrontal cortex of schizophrenia patients [11-13]. Schizophrenia patients showed reduced presynaptic DTNBP1 which was related to glutamatergic alterations in intrinsic hippocampal formation connections [11]. More recently, it has been established that reductions in protein in the brains of schizophrenia patients occurred in the dysbindin-1C isoform but not dysbindin-1A or -1B [14]. Studies also concluded that dysbindin-1C in the human brain is concentrated in the synapses [14]. Tang et al. [14] concluded that decreased dysbindin-1C in the dorsolateral prefrontal cortex may induce NMDA receptor hypofunction in fast-spiking interneurons contributing to the cognitive deficits of schizophrenia.

*DTNBP1 *is associated with other psychotic conditions. Association with *DTNBP1 *was found with bipolar disorder but only in a small subgroup characterised by the complication of psychotic features during episodes of mood disturbance [15]. Methamphetamine psychosis is clinically similar to schizophrenia [16,17] and a recent study hypothesised that *DTNBP1 *may also be associated with substance-induced psychoses [18]. Significant associations with methamphetamine psychosis and *DTNBP1 *variation was identified in a case-control study [18].

The aim of this study was to examine whether *DTNBP1 *variation shows genetic association with a range of psychiatric conditions, including schizophrenia, suggesting that changes in metabolism related to variations in DTNBP1 protein function underlies a common molecular defect in these conditions. A polymorphism in the human dopamine D2 receptor (*DRD2*) (rs6277) has previously been found to be associated with a number of psychiatric conditions or traits associated with psychopathology. The *DRD2 *polymorphism was first found to be associated with schizophrenia [19]. Since this study rs6277 has also been found to be associated with post-traumatic stress disorder (PTSD) [20], working memory ability [21], impulsivity [22] and alcohol dependence [23]. Like the *DRD2 *polymorphism, a common polymorphism in *DTNBP1 *may be associated with a range of psychiatric conditions. In a previous study, we found a polymorphism in *DTNBP1 *(rs9370822) that was strongly associated with schizophrenia [24]. It is also well known that anxiety and addictive disorders are common comorbidities of schizophrenia [25,26], so, to examine the importance of this *DTNBP1 *SNP in addictive and anxiety disorders, a number of psychiatric groups were genotyped for rs9370822.

## Methods

### Subjects

#### Controls

The control group consisted of 250 unrelated Caucasians (102 female and 148 male) with a mean age of 36.8 years (s.d. ± 12.8 years). The control group consisted mostly of medical and nursing staff recruited through hospitals, and university students and academic staff. Formal screening for psychological disorders was not undertaken in the control population. As such the controls represent an unselected control group.

#### Opiate dependence

A total of 120 unrelated Caucasian participants (50 female and 70 male) diagnosed as opiate dependent were recruited for this study, all of whom were being assessed for naltrexone treatment as outpatients of the Hospital Alcohol and Drug Services Unit of the Royal Brisbane and Women's Hospital. Participants had a mean age of 28.7 years of age. Approximately half of the participants were being managed on methadone prior to detoxification (47.2%) while the other half were on heroin (52.8%). Those on methadone had a mean dose of 48.1 milligrams (s.d. 30.5), with a range between 10-165 milligrams. The mean age of onset of heroin use was 22.4 years of age (s.d. 5.14), with a range between 15-43 years. Mean number of participant-reported detoxifications prior to this occasion was 3.5 (s.d. 3.3), with a range between 0-16 detoxifications. Cannabis was the most commonly concurrently used illicit substance reported by participants prior to treatment (52.5% reported use), followed by amphetamines (14.9% reported use).

#### PTSD

A total of 127 unrelated male Caucasian patients diagnosed with PTSD using Diagnostic and Statistical Manual of Mental Disorders-IV (DSM-IV) criteria were recruited through the Greenslopes Private Hospital for the study. All subjects were Vietnam combat veterans who had served in the Australian Armed Forces with a mean age of 52.3 years (s.d. ± 6.1 years). None were being treated with psychotropic medications. Patients were excluded from the study if they had a diagnosis of psychosis, bipolar disorder, obsessive-compulsive disorder, or organic brain syndrome including dementia. All subjects had sufficient comprehension of English and could understand the administered questionnaires. Patients were assessed for PTSD by a consultant psychiatrist or a senior psychiatric registrar using DSM-IV criteria. Furthermore, every patient exceeded the clinical cut-off score of 94 on the Mississippi Scale for combat-related PTSD [27]. Patient clinical history and demographic data including ethnic background were also obtained. After the procedure had been fully explained, all participants provided written informed consent. They were able to terminate their involvement at any time during the interview without prejudice. Institutional ethics approval was obtained from the Greenslopes Private Hospital.

#### Alcohol dependence

A total of 231 unrelated Caucasian (47 female and 184 male) alcohol dependent subjects were recruited from large public hospitals in Brisbane, Australia. The mean age of the group was 42.1 years (s.d. ± 10.8 years). All subjects were assessed by a clinical psychologist experienced in drug and alcohol dependence and met DSM-IV criteria of alcohol dependence disorder. All were inpatients and represented a spectrum of severity with a significant proportion (*n *= 65) of these patients being diagnosed with two or more alcohol related medical conditions such as pancreatitis, cirrhosis, hepatitis or peripheral neuropathy. Alcohol dependent patients were excluded from the study if they had dementia, delirium, psychosis, or any other condition that would affect their ability to provide informed consent.

#### Nicotine dependence

A total of 147 (68 male, 79 female) unrelated Caucasians with a mean age of 43.3 (s.d. ± 11.1 years) were recruited for this study through hospital and media advertisements. Participants were 18 years of age or older and had smoked for at least three years and were generally healthy despite currently smoking 15 cigarettes or more per day. All were motivated to reduce smoking and had the goal of eventual cessation. However, all participants had at least one serious but unsuccessful attempt at quitting in the previous 24 months. One hundred and thirty nine of the participants were administered the Fagerstrom test for nicotine dependence [28].

To minimise population stratification bias, both control and clinical subjects were recruited in the Brisbane region (a city of approximately 2 million inhabitants on the East Coast of Australia) and all were of British or European descent.

Ethics approval was obtained from all institutions involved and each participant gave written informed consent. This study was carried out in accordance with The Code of Ethics of the World Medical Association (Declaration of Helsinki).

### Genotyping

Oragene kits were used to extract DNA from saliva samples. Samples were genotyped using a homogeneous MassEXTEND (hME) Sequenom assay performed by the Australian Genome Research Facility. The hME assay is based on the annealing of an oligonucleotide primer (hME primer) adjacent to the SNP of interest. The addition of a DNA polymerase along with a mixture of terminator nucleotides allows extension of the hME primer through the polymorphic site and generates allele-specific extension products, each having a unique molecular mass. The resultant masses of the extension products are then analysed by matrix-assisted laser desorption/ionization time-of-flight mass spectrometry (MALDI-TOF MS) and a genotype is assigned in real time. The hME assay was performed in multiplex with up to 36 reactions in a single well. Genotyping failure rates varied between two and five percent in the different groups.

### Statistical analysis

A Pearson's χ^2 ^test was performed to identify statistical associations between alleles and genotype and schizophrenia status. Odds ratios (OR) were also calculated. Tests were performed on both genotype and allele data. Statistical tests were performed using the COMPARE2 program from the WinPepi suite of epidemiology programs [29] and SPSS version 16. Hardy-Weinberg equilibrium (HWE) was computed using Utility Programs for Analysis of Genetic Linkage [30]. The analysis of genotypes under a recessive model involved pooling the low-risk homozygotes and the heterozygotes and comparing frequencies with the high-risk homozygotes, i.e. OR > 1. Data were not corrected for multiple testing as only one SNP was tested in five entirely separate case groups.

## Results

In order to investigate the role of the *DTNBP1 *SNP, rs9370822, in a range of psychiatric conditions, it was genotyped in 250 controls, 127 PTSD subjects, 147 nicotine dependent subjects, 120 opiate dependent subjects and 231 alcohol dependent subjects. In addition to our previously reported association with schizophrenia, rs9370822 was found to be associated with PTSD, opiate dependence and nicotine dependence at the allele level but not with alcohol dependence (Table 1).

**Table 1:** Allele association of *DTNBP1 *SNP rs9370822

Sample set	**χ**^**2**^	*p*-value*	Odds ratio	95% CI
**Schizophrenia**^**†**^	9.883	0.002	1.61	1.19-2.17
**PTSD**	12.603	0.0004	1.78	1.28-2.50
**Nicotine dependence**	7.238	0.007	1.53	1.11-2.13
**Opiate dependence**	5.143	0.023	1.47	1.04-2.08
**Alcohol dependence**	1.957	0.162	1.22	0.92-1.61

* *p*-value determined by Pearson's χ^2 ^test

^† ^previously published data [24]

At the genotype level, rs9370822 was still associated with schizophrenia, PTSD, opiate dependence and nicotine dependence (Table 2). Individuals with PTSD were more than three times as likely to carry the CC genotype compared to the AA genotype. In addition, they were almost twice as likely to be heterozygous, suggesting a partially dominant mode of inheritance, ie the heterozygote OR was intermediate between the associated (CC) and non-associated (AA) genotypes (Table 2). Individuals with nicotine or opiate dependence were two and a half times as likely to carry the CC genotype compared to the AA genotype.

**Table 2:** Genotype association of *DTNBP1 *SNP rs9370822

Sample Set	Genotype counts			*p*-value*
	AA (%)	AC (%)	CC (%)	
control	113 (47.9)	101 (42.8)	22 (9.3)	
**Schizophrenia**^**‡**^	58 (37.2)	66 (42.3)	32 (20.5)	0.004 0.002^†^
Odds ratio	1.00	1.27	2.83	
*p*-value		0.57	0.002	

**PTSD**	36 (30)	62 (51.7)	22 (18.3)	0.002 0.0004^†^
Odds ratio	1.00	1.93	3.14	
*p*-value		0.02	0.00	

**Nicotine dependence**	46 (33.8)	70 (51.5)	20 (14.7)	0.022 0.007^†^
Odds ratio	1.00	1.70	2.23	
*p*-value		0.04	0.05	

**Opiate dependence**	45 (39.8)	47 (41.6)	21 (18.6)	0.04 0.027^†^
Odds ratio	1.00	1.17	2.40	
*p*-value		1.00	0.03	

**Alcohol dependence**	92 (41.63)	103 (46.6)	26 (11.76)	0.36 0.16^†^
Odds ratio	1.00	1.25	1.45	
*p*-value		0.51	0.49	

* *p*-value determined by Pearson's χ^2 ^test

^† ^*p*-value determined using the extended Mantel-Haenszel test for trend

^‡ ^previously published data [24]

Examination of the genotype odds ratios suggests that, like PTSD, a partially dominant mode of inheritance is operating for nicotine dependence. However, when compared to the low-risk homozygote, the odds ratio of the other two genotypes indicated that either a very weak partially dominant mode of inheritance or, more likely, a C-allele-recessive pattern of inheritance for schizophrenia and opiate dependence was present, i.e. both the heterozygote OR and the OR for the low-risk homozygote were approximately one. The rs9370822 SNP was found to be associated with schizophrenia and opiate dependence when analysed under a recessive model by pooling genotypes (schizophrenia recessive pattern of inheritance, *p *= 0.0017, OR = 2.51 95% CI 1.34 to 4.74; opiate dependence recessive pattern of inheritance, *p *= 0.014, OR = 2.22 95% CI 1.10 to 4.46).

Alcohol dependence was not found to be associated with rs9370822 at the genotype level or under any inheritance model. All sample groups were determined to be in Hardy-Weinberg equilibrium based on the respective genotype frequencies of each group (controls, χ^2 ^= 0.007, *p *= 0.93; PTSD, χ^2 ^= 0.272, *p *= 0.60; opiate dependence, χ^2 ^= 2.11, *p *= 0.15; nicotine dependence, χ^2 ^= 0.64, *p *= 0.42; alcohol dependence, χ^2 ^= 0.12, *p *= 0.73).

## Discussion

In order to identify a common molecular mechanism of disease etiology, *DTNBP1 *SNP, rs9370822, was chosen for analysis in a number of psychiatric conditions as we found it to be significantly associated with schizophrenia in a previous study [24]. To replicate our findings and examine the importance of this SNP in psychiatric disorders and potential brain functioning, we analysed a number of groups including addictive behaviours and anxiety disorders. In addition to schizophrenia, analysis of rs9370822 revealed associations with PTSD, opiate dependence and nicotine dependence at both the allele and genotype levels. Alcohol dependence was not found to be associated with rs9370822 in our study but association with rs9370822 cannot be ruled out as we only analysed 231 alcohol dependent cases and the *p*-value was approaching significance at both the allele and genotype level (Tables 1 and 2). While a strong effect of *DTNBP1 *was observed in four clinically distinct phenotypes the results should be treated with caution as there is a considerable difference in the gender proportions between cases and controls, for example the PTSD cohort were all male while 41% of controls were women. Also, because five different diseases were examined it is not surprising that there are also differences with regard to age (ranging from a mean age of 29 years in opiate dependent cases to 52 years in Vietnam veterans with PTSD).

Our schizophrenia study was the first to identify rs9370822 to be associated with a psychiatric condition [24]. The position of this SNP is likely to be a region for high schizophrenia susceptibility as three other SNPs (rs875462, rs760666 and rs7758659) flank rs9370822 and have been found to be significantly associated with schizophrenia [5,31]. We also found two other flanking SNPs (rs9370823 and rs4236176) to be associated with schizophrenia in a recent study [24]. Although the rs9370822 SNP is intronic it is possible that it is a functional SNP that affects RNA splicing or gene transcription. It could also be in linkage disequilibrium with a functional SNP. However a functional polymorphism in a nearby region that is associated with any psychiatric condition has yet to be identified.

The relationship between abnormal dysbindin function and addictive and anxiety disorders is unclear. Our findings may indicate that the rs9370822 polymorphism affects susceptibility to addictive and anxiety disorders by implication of glutamatergic neurotransmission or the dopamine system. We previously identified rs9370822 to follow a recessive pattern of inheritance with respect to schizophrenia risk [24] which is consistent with the recessive inheritance of dysbindin-1 gene deletion in the mouse model of schizophrenia [32]. In this study we also observed that opiate dependence appeared to follow a recessive pattern of inheritance. However, PTSD and nicotine dependence appeared to follow a partially dominant pattern. Given this, it is possible that rs9370822 results in a reduction of DTNBP1 function resulting in a recessive loss-of-function pattern in certain disorders, presumably because the loss of function from one allele is not sufficient to increase susceptibility to disease detectably but loss of function of both alleles does. In other disorders a haploinsufficiency mechanism is operating and results in a partially dominant mode of inheritance, i.e. loss of function in one allele increases susceptibility to disease but not as much as loss of function in both alleles.

It has also been reported that variation in *DTNBP1 *may affect the dopamine pathway as dysbindin overexpression decreases dopamine release and suppression of dysbindin expression increases dopamine release in the mouse *in vitro *[33]. A study has also found that dysbindin deficiency can increase the level of cell surface DRD2 and enhance DRD2 signalling [34].

## Conclusions

The *DTNBP1 *polymorphism identified in this study is possibly involved in psychiatric liability not only for schizophrenia but also for addictive and anxiety disorders. It is possible that *DTNBP1 *variation affects a common pathway involved in anxiety and addictive disorders. Whether this pathway involves dopamine or glutamate signalling or both, remains to be elucidated. As our participant numbers are limited our observations should be viewed with caution until they are independently replicated. However, the associations are unlikely to occur by chance due to sample size as five separate groups were analysed (including schizophrenia in a previous study) and four have been found to be significantly associated with rs9370822. Although this SNP is intronic, it has been found to be strongly associated with schizophrenia, PTSD, opiate and nicotine dependence. Future studies may explore whether this SNP is functional and what effect it has on *DTNBP1 *functioning in the brain.

Our findings are at odds with current knowledge as these conditions are generally regarded as separate clinical entities. However, our data suggests that these conditions may be different phenotypic expressions of the same fundamental molecular defect in either the dopamine or glutamate pathways, or both. Supporting this idea, a recent review of genome-wide association studies in schizophrenia [35] suggested that a pleiotropic mechanism may underlie the genetic overlap of schizophrenia with autism and with bipolar disorder.

## Competing interests

The authors declare that they have no competing interests.

## Authors' contributions

JV: Involved in conception and design, acquisition of data, analysed and interpreted data, drafted the article and approved final version. CS: Involved in conception and design, acquisition of data, critically revised article and approved final version. IH: Involved in conception and design, acquisition of data, analysed and interpreted data, critically revised article and approved final version. JC: Involved in acquisition of data, critically revised article and approved final version. BL: Involved in conception and design, acquisition of data, critically revised article and approved final version. RY: Involved in conception and design, acquisition of data, critically revised article and approved final version. CM: Involved in conception and design, acquisition of data, critically revised article and approved final version.
